# Mitochondrial non-coding RNA in nasopharyngeal carcinoma: Clinical diagnosis and functional analysis

**DOI:** 10.3389/fgene.2023.1162332

**Published:** 2023-03-23

**Authors:** Feng Wang, Xiaoyu Li, Cong Li

**Affiliations:** Department of Radiation Oncology, Chongqing University Cancer Hospital, Chongqing, China

**Keywords:** nasopharyngeal carcinoma, mitochondrial non-coding RNA, clinical diagnosis, functional analysis, feasible biomarkers

## Abstract

**Background:** Nasopharyngeal carcinoma is a common head and neck cancer with high incidence in Southeast Asia. Despite advances in treatment, the diagnosis of NPC remains a challenge due to its non-specific symptoms and high rate of false negatives. In this study, we aimed to identify novel non-coding RNAs (ncRNAs) as diagnostic biomarkers for NPC. Mitochondrial non-coding RNAs (mtio-ncRNAs) have been shown to play important roles in regulating various cellular processes. However, their specific functions and underlying mechanisms are largely unknown.

**Methods:** We investigated the expression and biological function of mtio-ncRNAs in the human NPC cell line C666-1. By using high-throughput sequencing, we identified several significantly expressed mtio-ncRNAs in C666-1 cells and analyzed their target genes and enriched pathways using tsRFUN.

**Results:** Our results showed that these significantly expressed mtio-ncRNAs mainly enriched in Cancer Gene Neighborhoods and targeted genes GCM1 and ACTG1. To validate the bioinformatics predictions, we synthesized two mtio-ncRNAs, t00846456 and t00048674, and transfected them into C666-1 cells. Our results showed that the expression of GCM1 was significantly increased by transfection of t00846456, while the expression of ACTG1 was significantly increased by transfection of t0048674. Additionally, the migration ability of the transfected cells was also enhanced.

**Discussion:** Our findings provide novel insights into the biological functions of mtio-ncRNAs and their potential applications in cancer diagnosis and treatment.

## Introduction

Nasopharyngeal carcinoma, a type of head and neck cancer, is a common malignancy in Southeast Asia, with a high incidence rate in Southern China (Chua et al., 2016). Despite advances in diagnosis and treatment, the 5-year overall survival rate for NPC patients remains low due to the high frequency of locoregional relapse and distant metastasis (Szymańska, 2019). The etiology of NPC is complex, involving genetic susceptibility, environmental exposure, and viral infection. Epstein-Barr virus (EBV) infection has been identified as a crucial risk factor for NPC development (Tsao et al., 2017). Understanding the molecular mechanisms underlying NPC pathogenesis is crucial for the development of effective therapeutic strategies and the improvement of patient outcomes. In recent years, significant progress has been made in our understanding of the role of EBV in NPC, including the characterization of EBV-encoded oncogenes, the identification of host genetic susceptibility factors, and the development of EBV-targeted therapies (Young and Dawson, 2014). Diagnosis and treatment of NPC remain a challenge, with a need for more effective and less invasive approaches. The current standard for NPC diagnosis is a combination of imaging techniques, biopsy, and serological testing (Tabuchi et al., 2011). Treatment options for NPC include radiotherapy, chemotherapy, and surgery, with the choice of treatment depending on the stage and location of the disease. Despite advances in treatment, the outcome for NPC patients remains unsatisfactory, with a high frequency of treatment resistance and relapse (Zhang et al., 2013).

In addition to viral and host factors, recent studies have revealed a crucial role for small non-coding RNA molecules in NPC pathogenesis. Mitochondrial-derived small non-coding RNAs (mito-ncRNAs), a class of small RNA molecules derived from the mitochondria, have been shown to play important roles in a variety of cancer types, including regulation of cellular metabolism, apoptosis, and oxidative stress. However, the role of mito-ncRNAs in NPC has yet to be fully explored. Understanding the role of mito-ncRNAs in NPC pathogenesis may provide new insights into the biological mechanisms underlying NPC development and progression and may lead to the discovery of novel therapeutic targets. In this study, we aim to investigate the role of mito-ncRNAs in NPC. We analyzed miRNA sequencing data from serum samples of NPC patients in a public dataset and re-analyzed the data to obtain the expression of mito-ncRNAs. Based on this expression, we constructed a diagnostic model and validated it using serum samples collected from NPC patients. Moreover, we also over-expressed two particularly significant mito-ncRNAs and predicted and validated their target genes. Our study provides novel insights into the potential use of mito-ncRNAs as non-invasive diagnostic markers for NPC and may aid in improving our understanding of NPC pathogenesis.

## Methods

### Datasets

We collected serum miRNA-seq samples of nasopharyngeal cancer and normal groups from the Gene Expression Omnibus dataset, including 6 nasopharyngeal cancer patients and 6 normal controls, with the GEO ID of GSE163867 (Zheng et al., 2021). In addition, we independently collected serum samples from 13 nasopharyngeal cancer patients and 11 normal patients from March 2020 to December 2022, the clinical data can be found in the Supplementary Table S1. Furthermore, we obtained oral informed consent from the patients and passed ethical review.

### Extraction of Mito-ncRNA expression from miRNA-seq data

We extracted Mito-ncRNA expression from the miRNA-seq data following the methods described in the published article (Yu et al., 2022). Briefly, the miRNA-seq data was first downloaded from the SRA tool and then subjected to adapter trimming using the nf-core/smrnaseq pipeline in Nextflow. After trimming with the pipeline in Nextflow, the sequences were aligned to the mitochondrial genome to obtain Mito-ncRNA expression data. The missing values were imputed using MetImp 1.2 (Wei et al., 2018).

### Prediction of Mito-ncRNA functions

Twenty-two mito-ncRNAs that were differentially expressed (Log2FoldChange (Cancer/Normal) > 1 and *p*-value <0.05) were selected from the sequencing data. The functional predictions of these mito-ncRNAs were made using the tsRFun software (Wang et al., 2022). Relationships between these mito-ncRNAs and mRNAs were analyzed using data from Fetch, CLEAR, CLIP, and CLASH and functional enrichment analyses were performed using gene ontology annotation and cancer gene neighborhood analysis.

### Cell culture

The C666-1 cell line was derived from undifferentiated nasopharyngeal carcinoma (NPC) and consistently carries the Epstein-Barr virus (EBV) in long-term cultures (Cheung et al., 1999). The cells were maintained in Dulbecco’s modified Eagle’s medium (DMEM) supplemented with 10% fetal bovine serum (FBS), 100 U/mL penicillin, and 100 μg/mL streptomycin in a humidified incubator at 37°C with 5% CO2. Before each experiment, cells were seeded in 6-well plates and allowed to grow until 70%–80% confluence was reached. The medium was then replaced with fresh medium for the experiment.

### RT-PCR

RT-PCR was performed to quantify gene expression levels of the target genes. Total RNA was extracted from the samples using Trizol reagent (Invitrogen, Carlsbad, CA, United Stated) according to the manufacturer’s instructions. cDNA was synthesized from 1 μg of total RNA using a reverse transcription kit (Takara, Dalian, China) and then used as the template for PCR amplification. The RT-PCR reactions were performed in a final volume of 20 μL, containing 10 μL of 2x TaqMan universal PCR Master Mix, 0.5 μL of each 20 μM forward and reverse primers, and 2 μL of cDNA template. The thermal cycling conditions were as follows: 50°C for 2 min, 95°C for 10 min, followed by 40 cycles of 95°C for 15 s and 60°C for 1 min. The primers for RT-PCR are listed in the Supplementary Table S3. The relative expression levels of the target genes were quantified by the 2^−ΔΔCT^ method and normalized to the GAPDH control gene. t00846456 sequence: ATCCTGCTCACAGCGCCA and t00048674 sequence: AGT​GGT​AGA​ATT​CTC​GCC​TGC.

### Western blot

The Western blot analysis was performed to assess the protein expression levels of GCM1 and ACTG1, as well as a reference protein, GAPDH. The cell lysates were resolved by SDS-PAGE and transferred onto nitrocellulose membranes. The membranes were blocked with 5% non-fat dry milk in TBST for 1 h at room temperature and then incubated overnight with primary antibodies specific to GCM1, ACTG1, and GAPDH at 4°C. The following day, the membranes were washed three times with TBST for 5 min each and then incubated with horseradish peroxidase (HRP)-conjugated secondary antibody for 1 h at room temperature. The protein signals were detected using an enhanced chemiluminescence (ECL) system. The protein expression levels were quantified by normalizing the protein bands to the reference protein, GAPDH.

### Transfection

We used the synthesized mitochondrial non-coding RNA from Takara, (Dalian, China) for overexpression analysis in our study. The mito-ncRNA was resuspended in Opti-MEM® I Reduced Serum Medium (Invitrogen, Carlsbad, CA, United States) at a final concentration of 20 nM. The transfection was performed using Lipofectamine 3,000 transfection reagent (Invitrogen) according to the manufacturer’s instructions. In brief, the knockdown plasmid solution was mixed with the Lipofectamine 3,000 solution in a 1:1 ratio and incubated at room temperature for 20 min. The transfection mixture was then added to the cells in culture medium. The cells were harvested for analysis 48 h after transfection.

### Transwell

The Transwell migration assay was performed to evaluate the effect of the identified mito-ncRNAs on cell migration. The C666-1 cell line was seeded into the upper compartment of a transwell insert (8 μm pore size, Corning Inc., NY, United States) at a density of 2 × 105 cells per well. The lower compartment was filled with complete growth medium as a chemoattractant. After 24 h of incubation at 37°C, the cells that had migrated through the membrane were fixed with 4% paraformaldehyde for 30 min and stained with 0.1% crystal violet for 30 min. The migrated cells were then counted under a light microscope (Nikon, Tokyo, Japan) at ×200 magnification. The experiment was performed in triplicate.

## Results

### Screening of gene expression and key mitochondrial non-coding RNAs

**FIGURE 1:**
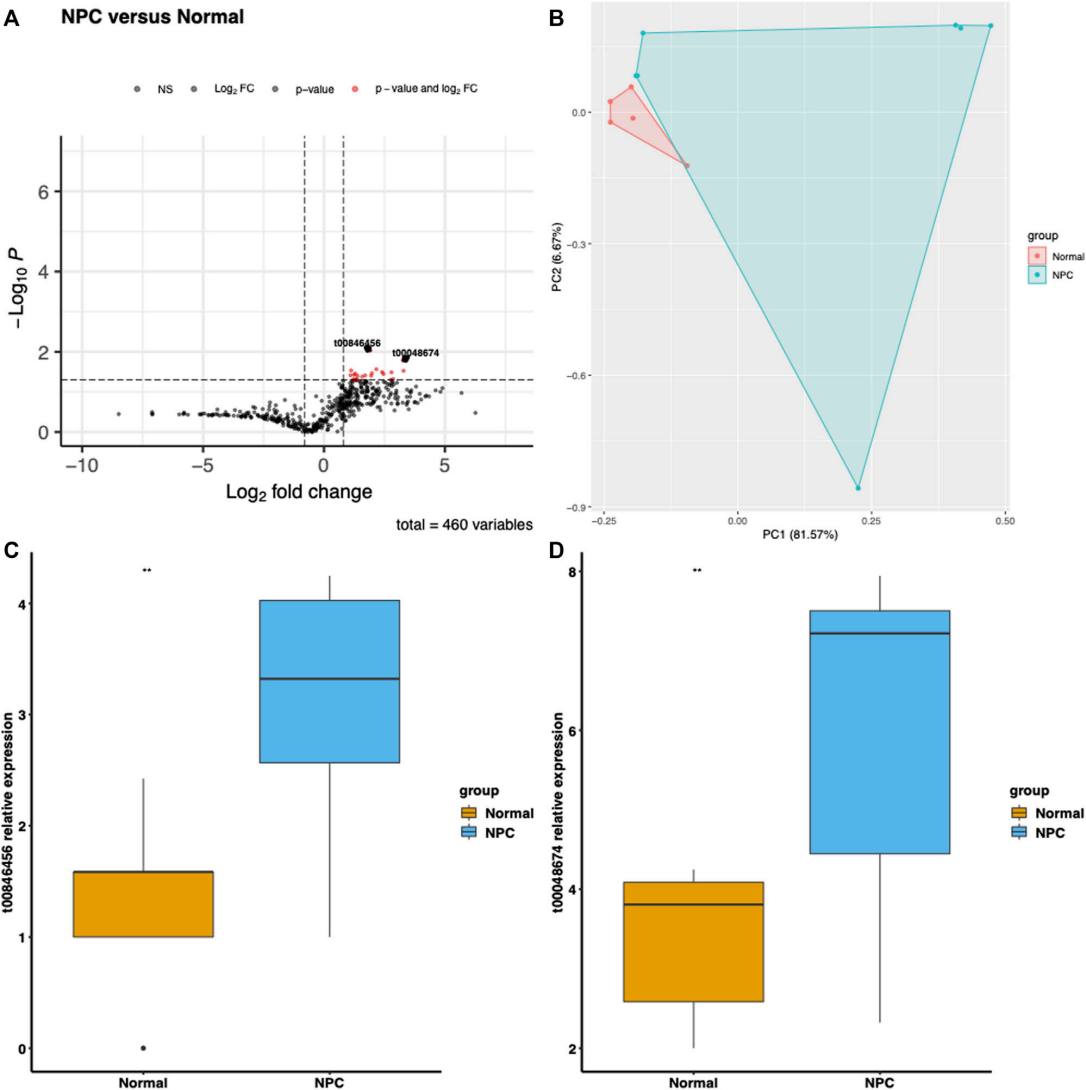
Differential gene expression in Nasal Pharyngeal Cancer and Normal groups. **(A)** Volcano plot displaying the log2 fold change and -log10 *p*-value for each gene in the dataset. Comparing the expression levels of nasal pharyngeal cancer and normal groups for two significantly expressed mitochondrial-derived small RNAs, t00048674 and t00846456, which were selected based on a Log2FoldChange >1 and *p*-value <0.05 criterion. **(B)** Principal component analysis (PCA) plot illustrating the differentiation between Nasal Pharyngeal Cancer and Normal samples based on gene expression levels. Each point represents a sample, with Nasal Pharyngeal Cancer samples indicated in red and Normal samples in blue. The plot shows the first two principal components (PC1 and PC2), which account for the majority of the variation in the data. **(C)** Boxplot of t00846456 showing the distribution of expression levels in Nasal Pharyngeal Cancer and Normal groups. The expression level of t00846456 is significantly upregulated in the Nasal Pharyngeal Cancer group. **(D)** Boxplot of t00048674 displaying the distribution of expression levels in Nasal Pharyngeal Cancer and Normal groups. The expression level of t00048674 is also significantly upregulated in the Nasal Pharyngeal Cancer group.

We extracted the expression levels of mitochondrial derived small RNAs from the GSE163867 public dataset. Figure 1A shows the *t*-test results of the mitochondrial derived small RNAs in the serum of NPC group and normal group, including *p* values and Fold change. 22 significant mitochondrial derived small RNAs (Supplementary Table S2) were screened based on *p* < 0.05 and log2FC (Cancer/Normal) > 1. The two most significantly expressed mitochondrial derived small RNAs were t00846456 and t00048674. PCA analysis was performed on these 22 significant mtio-ncRNAs and revealed clear separation between the NPC group and the Control group (Figure 1B). The expression levels of these two significant mtio-ncRNAs were displayed in boxplots in Figures 1C, D.

### Establishing a diagnostic model for mitochondrial derived small RNAs and validation of the model

**FIGURE 2:**
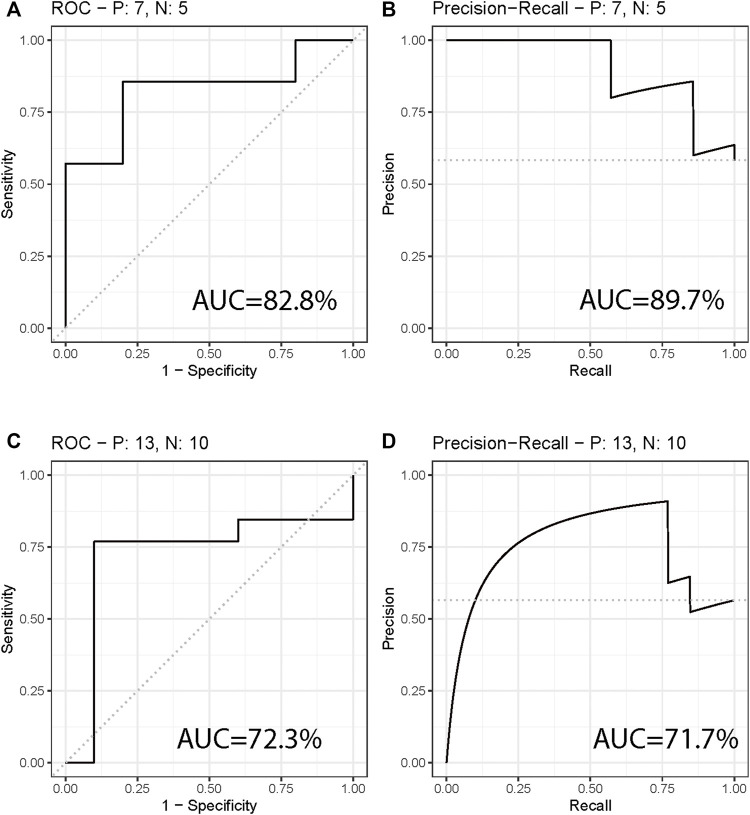
Diagnostic Model for Differentiating Nasal Pharyngeal Cancer from Normal. **(A,B)** ROC curve and PR curve showing the performance of the diagnostic model in differentiating Nasal Pharyngeal Cancer and Normal groups. The areas under the ROC curve (AUC) are 82.9% and 89.7% respectively. **(C,D)** ROC curve and PR curve showing the performance of the diagnostic model in an independent validation set. The AUC areas are 72.3% and 71.7% respectively.

The two most significant mtio-ncRNAs were integrated into a random forest model to establish a diagnostic model for NPC. The performance of the model was evaluated using ROC and PR curves, and the results (Figures 2A, B) showed that the ROC and PR areas for NPC and control groups were 82.9% and 89.7% respectively. Additionally, the diagnostic model was validated using an independent dataset collected, with ROC and PR areas of 72.3% and 71.7% respectively (Figures 2C, D).

### Functional prediction and experimental validation of mitochondrial derived small RNAs

**FIGURE 3:**
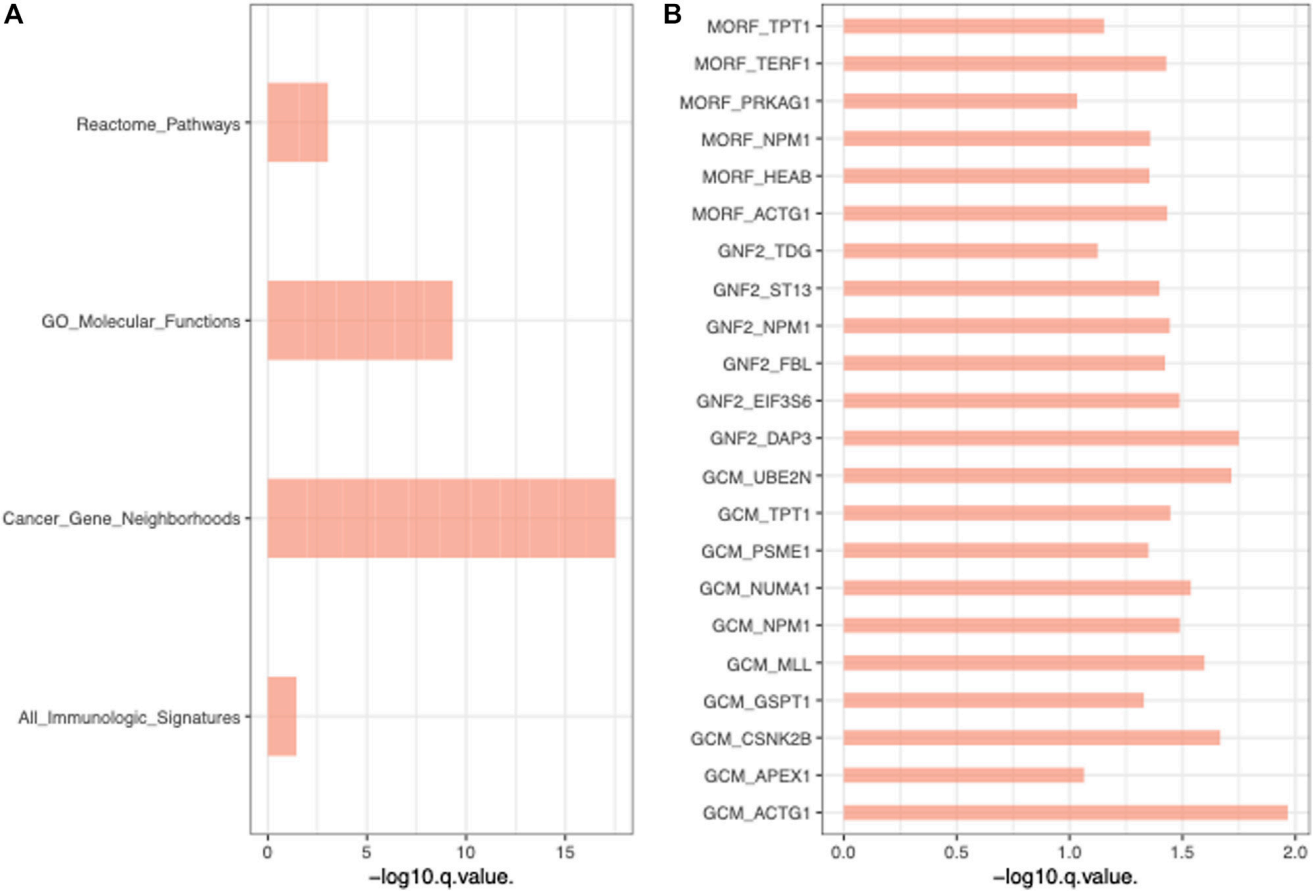
Functional Analysis of Significant mtio-ncRNAs Target Genes and Enriched Pathways. **(A)** Enriched pathways of all pathways, with Cancer Gene Neighborhoods pathway being the most significant. **(B)** The Cancer Gene Neighborhoods pathway, with GCM1 and ACTG1 as the two most significant genes in the pathway.

**FIGURE 4:**
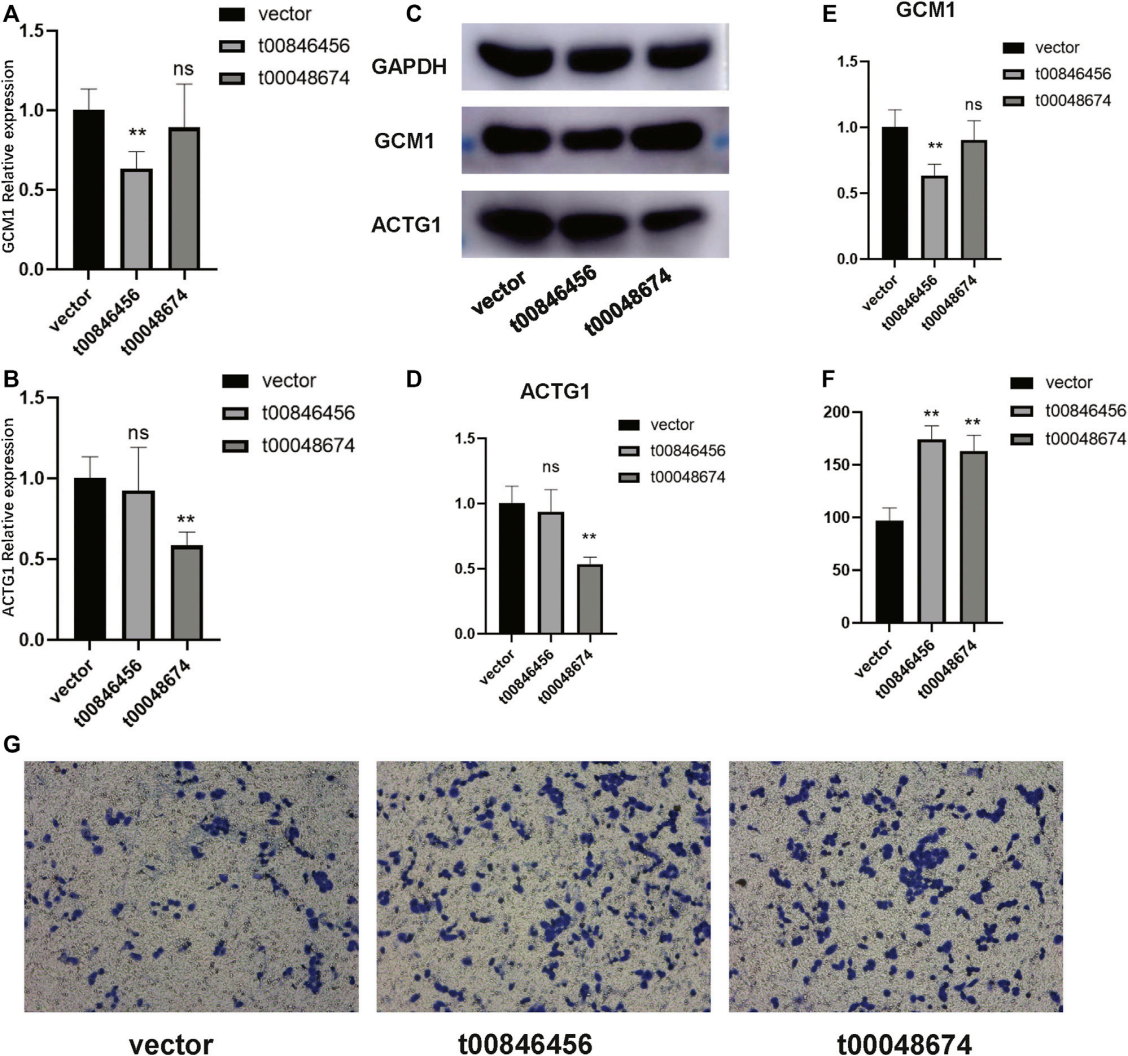
Validation of the experimentally confirmed target genes predicted from the bioinformatics analysis. **(A)** The expression of GCM1 significantly decreased after transfection with t0048674 knock down plasmid. **(B)** The expression of ACTG1 significantly decreased after transfection with t00846456 knock down plasmid. **(C–E)** Western blot results demonstrating that after transfection with t0048674 knock down plasmid, the corresponding ACTG1 protein was significantly decreased, and after transfection with t00846456 knock down plasmid, the corresponding GCM1 protein was significantly decreased. **(F)** A bar graph showing that the migration ability of cells transfected with over-expression of t00846456 and t0048674 significantly increased. **(G)** A trans well graph showing that the migration ability of cells significantly increased after transfection with over-expression of t00846456 and t0048674.

To further validate the functions of these significant mtio-ncRNAs, we used tsFUN to analyze their target genes and enriched pathways (Figures 3A, B). Results showed that these significantly expressed mtio-ncRNAs mainly enriched the Cancer Gene Neighborhoods pathway, and further analysis of the Cancer Gene Neighborhoods pathway revealed that the main target genes were GCM1 and ACTG1 genes. To further validate the results of bioinformatics prediction, we knockdown mt-ncRNAs, t00846456 and t0048674, and transfected them into C666-1 cells. Figures 4A, B respectively show the expression of GCM1 and ACTG1 after transfection of t00846456 and t0048674. The results showed that the expression of ACTG1 significantly decreased after transfection of t00048674, while the expression of GCM1 significantly decreased after transfection of t00846456, with the same results observed in protein expression (Figure 4D). After overexpression of t00846456 and t00846456, the migration ability of cells transfected with t00846456 and t00048674 increased (Figures 4C, E).

## Discussion

In this study, we aimed to identify novel diagnostic biomarkers and target genes for NPC through the analysis of miRNA-ncRNA interactions. By using a combination of bioinformatics and experimental validation, we found two miRNAs (t00846456 and t00048674) to be significantly overexpressed in NPC tissues compared to the control group. Further analysis revealed that these miRNAs mainly enriched the Cancer Gene Neighborhoods pathway, with GCM1 and ACTG1 as the main target genes. Synthetic overexpression of these two miRNAs *in vitro* led to the upregulation of GCM1 and ACTG1 expression, as well as increased cell migration ability. GCM1, a Glial Cells Missing 1 transcription factor, has been implicated in tumorigenesis, angiogenesis, and invasion of various cancers (Liang et al., 2010; Matsuura et al., 2011; Li and Roberson, 2017). On the other hand, ACTG1, a β-actin gene, is involved in cell migration and invasion (Richter et al., 2020; Xiao et al., 2021). These findings suggest that these two genes may play a role in the development and progression of NPC.

The results of our diagnostic model, which was built using the random forest algorithm and validated using an independent dataset, showed high diagnostic accuracy with ROC and PR areas of 82.9% and 89.7% for the training set and 72.3% and 71.7% for the validation set, respectively. These results suggest that these two miRNAs have potential as diagnostic biomarkers for NPC.

However, our study also has some limitations. The sample size of our validation set was relatively small, which may limit the generalizability of our results. In addition, our study was limited to a single cell line, and further validation in a larger and more diverse patient cohort is necessary to confirm the utility of these miRNAs as diagnostic markers.

In conclusion, our study provides novel insights into the role of miRNA-ncRNA interactions in NPC and highlights the potential diagnostic value of these two miRNAs as non-invasive biomarkers for NPC. Further studies are necessary to confirm these findings and to fully understand the underlying mechanisms of NPC development and progression.

## Data Availability

The datasets presented in this study can be found in online repositories. The names of the repository/repositories and accession number(s) can be found in the article/Supplementary Material.
