# Supplementary material for: Mitochondrial non-coding RNA in nasopharyngeal carcinoma: Clinical diagnosis and functional analysis
Source: Front Genet. 2023 Mar 23;14:1162332. doi: 10.3389/fgene.2023.1162332 (PMC10076541; doi:10.3389/fgene.2023.1162332)
Supplement: Supplementary file 1 [file DataSheet1.docx]

Table 1. The clinical and pathological information of the patients in this study

| Number | Age | Sex | Stage | T | N | M |
| --- | --- | --- | --- | --- | --- | --- |
| 1 | 73 | MALE | III | T3 | N0 | M0 |
| 2 | 52 | FEMALE | II | T1 | N1 | M0 |
| 3 | 47 | MALE | III | T2 | N2 | M0 |
| 4 | 47 | MALE | IV | T3 | N2 | M0 |
| 5 | 67 | MALE | I | T1 | N0 | M0 |
| 6 | 36 | FEMALE | I | T1 | N1 | M0 |
| 7 | 64 | FEMALE | III | T2 | N2 | M0 |
| 8 | 48 | MALE | IVA | T3 | N3 | M0 |
| 9 | 60 | FEMALE | IVB | T2 | N2 | M1 |
| 10 | 53 | FEMALE | II | T1 | N2 | M0 |
| 11 | 58 | MALE | II | T2 | N0 | M0 |
| 12 | 57 | FEMALE | III | T2 | N1 | M0 |
| 13 | 68 | MALE | IVB | T2 | N1 | M1 |

Table2. Significantly Expressed 22 Mitochondrial-Derived Small RNAs in Nasopharyngeal Cancer and Normal Groups

|  | avg_1 | avg_2 | log2FC | pvals |
| --- | --- | --- | --- | --- |
| t00846456 | 1.52306006 | 3.44076241 | 1.91770235 | 0.00927219 |
| t00048674 | 3.5849625 | 6.84906994 | 3.26410744 | 0.01647867 |
| t00629158 | 2.40653667 | 4.57020268 | 2.16366601 | 0.02710736 |
| t00063439 | 4.60880924 | 5.73180389 | 1.12299465 | 0.02908377 |
| t00276150 | 2.43295941 | 5.72012208 | 3.28716268 | 0.02969582 |
| t00032779 | 3.48542683 | 5.88648047 | 2.40105364 | 0.03184846 |
| t00682834 | 0.84799691 | 3.63542274 | 2.78742583 | 0.03239714 |
| t00002401 | 6.54843663 | 8.52691836 | 1.97848174 | 0.0340842 |
| t00101489 | 2.76553475 | 4.05522045 | 1.2896857 | 0.03484511 |
| t00192167 | 1.5290713 | 3.98810035 | 2.45902905 | 0.03571413 |
| t00038306 | 3.9068906 | 5.14684139 | 1.23995079 | 0.03749455 |
| t00034777 | 4.24762376 | 5.34335706 | 1.0957333 | 0.03841084 |
| t00050367 | 3.13750352 | 4.83650127 | 1.69899774 | 0.03861944 |
| t00029788 | 4.49825087 | 6.44531051 | 1.94705964 | 0.03955865 |
| t00013808 | 4.23266076 | 5.61470984 | 1.38204909 | 0.03956961 |
| t00018982 | 4.64385619 | 6.22055108 | 1.57669489 | 0.04168543 |
| t00003359 | 5.81249823 | 8.6714147 | 2.85891647 | 0.04734764 |
| t00054575 | 3.0499792 | 4.37145929 | 1.32148009 | 0.04829063 |
| t00034504 | 4.26303441 | 5.64385619 | 1.38082178 | 0.04910299 |
| t00049514 | 2.84398384 | 4.05062607 | 1.20664223 | 0.04920343 |
| t00157168 | 2 | 4.75915583 | 2.75915583 | 0.04942531 |
| t00708718 | 2.5849625 | 3.82578563 | 1.24082313 | 0.04952094 |

Table 3 Primer list

| id | Primer |
| --- | --- |
| GCM1_Forward Sequence | AGTGAACACAGCACCTTCCTCC |
| GCM1_Reverse Sequence | TTGGACGCCTTCCTGGAAAGAC |
| ATCG1_Forward Sequence | CACCATTGGCAATGAGCGGTTC |
| ATCG1_Reverse Sequence | AGGTCTTTGCGGATGTCCACGT |
